# A Vision-Based Method for Determining Aircraft State during Spin Recovery

**DOI:** 10.3390/s20082401

**Published:** 2020-04-23

**Authors:** Tomasz Kapuscinski, Piotr Szczerba, Tomasz Rogalski, Pawel Rzucidlo, Zygmunt Szczerba

**Affiliations:** 1 Department of Computer and Control Engineering, Faculty of Electrical and Computer Engineering, Rzeszow University of Technology, W. Pola 2, 35-959 Rzeszow, Poland; 2 Department of Avionics and Control Systems, Faculty of Mechanical Engineering and Aeronautics, Rzeszow University of Technology, Aleja Powstancow Warszawy 12, 35-959 Rzeszow, Poland; psz@prz.edu.pl (P.S.); orakl@prz.edu.pl (T.R.); pawelrz@prz.edu.pl (P.R.); 3 Department of Aerodynamics and Fluid Mechanics, Faculty of Mechanical Engineering and Aeronautics, Rzeszow University of Technology, Aleja Powstancow Warszawy 12, 35-959 Rzeszow, Poland; zygszcze@prz.edu.pl

**Keywords:** aircraft spin recovery, aircraft spin phase detection, computer vision, image analysis, keypoints matching

## Abstract

This article proposes a vision-based method of determining in which of the three states, defined in the spin recovery process, is an aircraft. The correct identification of this state is necessary to make the right decisions during the spin recovery maneuver. The proposed solution employs a keypoints displacements analysis in consecutive frames taken from the on-board camera. The idea of voting on the temporary location of the rotation axis and dominant displacement direction was used. The decision about the state is made based on a proposed set of rules employing the histogram spread measure. To validate the method, experiments on flight simulator videos, recorded at varying altitudes and in different lighting, background, and visibility conditions, were carried out. For the selected conditions, the first flight tests were also performed. Qualitative and quantitative assessments were conducted using a multimedia data annotation tool and the Jaccard index, respectively. The proposed approach could be the basis for creating a solution supporting the pilot in the process of aircraft spin recovery and, in the future, the development of an autonomous method.

## 1. Introduction

An aircraft spin is a specific flight condition that occurs in all types of aviation. In this state, the trajectory has the characteristic form of a spiral line. Specific actions are required to recover from the spin and to avoid the plane crash. There are two types of spin flat and steep. In the flat one, the plane’s pitch angle is less than 45 degrees, whereas, in the steep one, it is between 45 and 90 degrees. This article concerns the steep case when the pilot sees the image of the rotating earth and can return to the normal state by appropriate actions.

**Figure 1:**
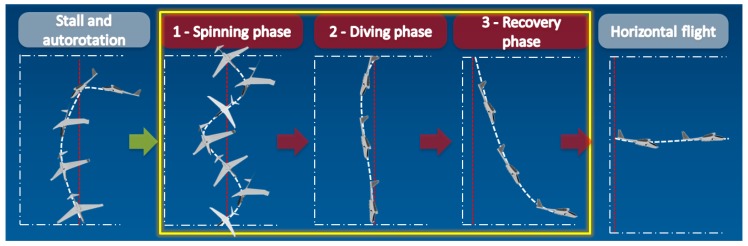
Phases of flight during spin recovery.

When recovering from a spin, three phases of flight are defined: rotation, diving, and recovery (see Figure 1).

Each of them requires a different pilot’s action. This paper proposes a vision-based method of identification in which of these phases an aircraft is.

The presented solution could be part of the pilot assistance system and, in the future, the basis of the method for automatic spin recovery. The spin recovery procedure does not seem difficult, but the problem is the spatial disorientation of the pilot [1,2,3]. According to the Air Safety Institute of Aircraft Owners and Pilots Association (AOPA), in the years 2000–2014, 30 percent of stall related accidents in commercial flights caused fatalities [4]. Therefore such a solution would significantly improve safety.

Research on the spin phenomenon, conducted since the beginning of the 20th century, concerns the dynamics of aircraft in this state [5,6,7,8,9], or recovery procedures [10,11,12,13,14,15]. Control algorithms are also being developed to enable automatic spin recovery but mainly for military or experimental applications [16,17,18,19]. However, these methods assume that we can precisely determine the instantaneous state of the aircraft. Proposed solutions are mainly based on inertial sensors, which measure the aircraft state indirectly, for example, through the analysis of angular velocities. Such analysis may sometimes lead to ambiguous results. Therefore, direct measurement using a vision sensor is a desirable and innovative solution.

Vision systems are increasingly used in aviation to detect threats from intruder objects appearing in the operating space [20,21,22], or in navigation [23,24,25]. There are also known solutions that use cameras for spin analysis. Aircraft models are observed in specially designed wind tunnels [26,27]. However, these are solutions in which the view from the perspective of an external observer is used and they are designed to know how the different aircraft structural elements influence on the spin character.

Several works regarding the estimation of flying object state can be found in the literature. In [28], the attitude of the aircraft model placed in the vertical wind tunnel is measured using the stereo vision method. To achieve high robustness markers are attached to the surface of the plane. A vision system for a helicopter model six degrees of freedom pose estimation is proposed in [29]. It uses a pan/tilt/zoom ground camera and another small onboard imager. The algorithm is based on tracking of five colored blobs placed on the aircraft and a single marker attached to the ground camera. A system for precision projectiles roll and pitch estimation by interpreting data from a strapped-down, forward-facing imager is described in [30]. The solution is based on the horizon detection algorithm, employing the Hough transform and an intensity standard deviation method. Robust, real-time state estimation of micro air vehicles is proposed in [31]. The method is based on tracking the feature points, such as lines and planes, and the implicit extended Kalman filter. According to the authors, a vision-based estimation is an attractive option, especially in urban environments. In [32], a vision-based method of aircraft approach angle estimation is presented. Several sequential images are used to determine the horizon and the focus-of-expansion, and then to derive the angle value. A glider control system with vision-based feedback is presented in [33]. The proposed navigation algorithm allows for reaching the predetermined location. The position of the target in the image is determined by integrating the pixel intensities across the image and performing a cascade of feature matching functions. Then a Kalman filter is used to estimate attitude and glideslope.

The approach proposed in this work is original. According to the authors’ best knowledge, no other studies are published in which images from the on-board camera are used to determine the condition of the aircraft in the spin. An additional advantage of the vision-based method is passive measurement. It also does not require significant modification of the aircraft structure.

The main novelty and contributions of this paper are: (i) unique application based on the vision sensor only, (ii) proposal of mappings from the image sequence (space-time domain) to the parameter space to determine the rotation axis and the movement direction by voting technique and maxima detection in the accumulator matrices, (iii) analyzing of the accumulator matrices using the histogram spread measure, (iv) a set of rules proposal to estimate the aircraft spinning state, and (v) creating a unique dataset, annotated by a human expert, containing various simulation data as well as preliminary flight recordings, and making it available to the research community for fair comparisons, (vi) experimental verification of the method using data from the simulator and real recordings in-flight tests, and (vii) original application of the multimedia data annotation package—ELAN for qualitative analysis of results.

The structure of this paper is as follows. Section 1 defines the problem, gives the research background and relevant references. Section 2 describes the proposed method. Experiments are presented in Section 3. Section 4 concludes the paper and indicates further works.

## 2. Method

**Figure 2:**
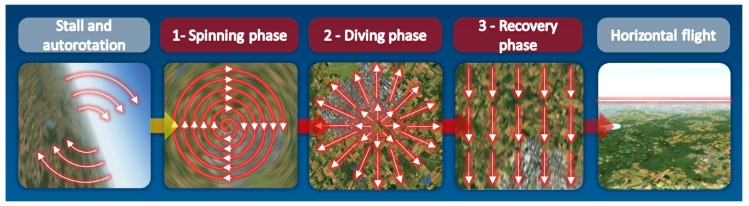
Keypoints displacements in diffrent phases.

The general idea of the proposed solution is to search for the corresponding keypoints in successive video frames and to conclude about the temporary state of the aircraft during spin recovery, based on the analysis of the displacement of these points (see Figure 2). Details of the method are presented in Section 2.1, Section 2.2, Section 2.3, and Section 2.4.

### 2.1. Keypoints Detection and Matching

The concept of keypoints is widely used in computer vision for the tasks of object recognition, image registration, or 3D reconstruction. These points are related to local image features that persist over some period. Each keypoint is associated with the so-called descriptor. It is a set of distinctive features that can be used to search for corresponding points in different images. Several keypoints detectors and their descriptors have been developed, e.g., scale-invariant feature transform (SIFT) [34], gradient location and orientation histogram (GLOH) [35], speeded up robust features (SURF) [36], or local energy-based shape histogram (LESH) [37].

During spin recovery, the sizes of the objects, visible in images from an on-board camera, change quickly and randomly. Therefore, a scale-independent detector was considered, and finally, SURF was selected because it is faster than SIFT. The SURF keypoints are robust against different image transformations. Their descriptors ensure repeatability and distinctiveness [38]. Interest points are found at different scales using the multi-resolution pyramid technique. Therefore they are rotation and scale-invariant, which is essential in the considered problem.

**Figure 3:**
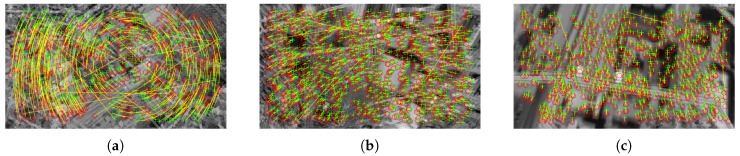
SURF keypoints determined at t−1 (red circles) and *t* (green crosses) connected by displacements (yellow segments) plotted on t−1 and *t* frames superimposed using alpha blending for: (**a**) spinning phase, (**b**) diving phase, (**c**) recovery phase.

Feature matching can be done by calculation of the pairwise distance between descriptors. However, to speed up the processing, an approximate nearest neighbor search was applied [39]. Let $P_{t-1}$ and $P_{t}$ denote sets of *N* corresponding keypoints detected at the moment t−1 and *t*:

(1) $$\begin{array}{cc} P_{t-1} & =\{p_{t-1}=\left(x_{t-1},y_{t-1}\right);p_{t-1}\equiv p_{t}\}\\ P_{t} & =\{p_{t}=\left(x_{t},y_{t}\right);p_{t-1}\equiv p_{t}\} \end{array}$$

where ≡ denotes the correspondence of points. The corresponding keypoints detected in two successive images acquired during spin recovery are shown in Figure 3.

### 2.2. Removing Faulty Keypoints Matchings

**Figure 4:**
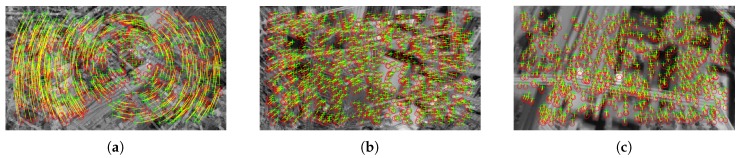
SURF keypoints from Figure 3 after removing faulty matching for: (**a**) spinning phase, (**b**) diving phase, (**c**) recovery phase.

As shown in Figure 3, some displacements diverge from the others. They are much longer, and their directions “do not match” the visible change trend. They are the result of incorrect keypoints matching and may adversely affect the further analysis. Therefore, we filter out the points that do not satisfy the following criterion:

(2) $$|r_{t}-\mu_{t}|\leq k\sigma_{t}$$

where $r_{t}=\sqrt{{\left(x_{t}-x_{t-1}\right)}^{2}+{\left(y_{t}-y_{t-1}\right)}^{2}}$, $\mu_{t}=\frac{1}{N}\sum_{i=1}^{N}r_{t,i}$, $\sigma_{t}=\sqrt{\frac{1}{N}\sum_{i=1}^{N}{\left(r_{t,i}-\mu_{t}\right)}^{2}}$, and *k* is a parameter of the method. This procedure is applied to increase the robustness of the method. The corresponding keypoints after removing faulty matchings are shown in Figure 4.

### 2.3. Voting

**Figure 5:**
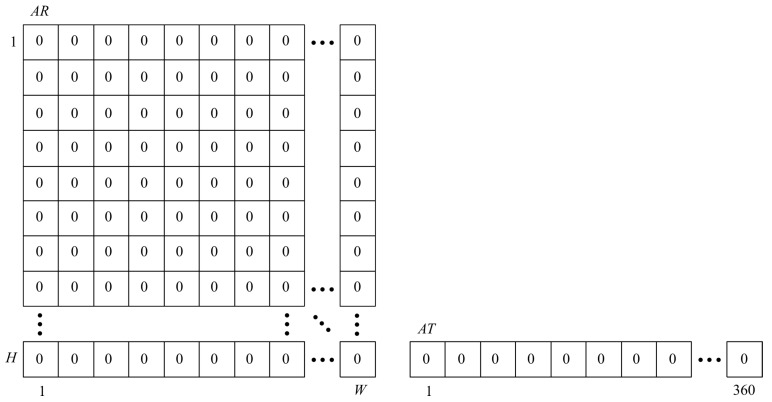
Accumulator matrices.

To find the temporary position of the rotation axis and the dominant direction of the shift vector, a voting scheme, similar to that used in the Hough transform, was applied [40]. Two so-called accumulator matrices: AR (dimAR=W×H) and AT (dimAT=1×360), were created and filed with zeros. *W* and *H* denote the image width and height, respectively (Figure 5).

**Figure 6:**
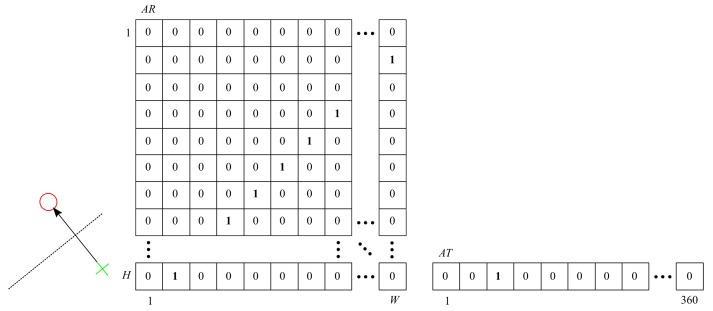
AR and AT after taking into account the “votes” of one pair of corresponding points.

Each vector ${\vec{r}}_{t}=p_{t}-p_{t-1}$ “votes” for all possible positions of the hypothetical rotation axis, according to the idea presented in Figure 6.

The AR cells, through which the $\bar{p_{t}p_{t-1}}$ segment bisector passes, are incremented.

(3) $$\forall_{{\vec{r}}_{t}}AR\left[x,y\right]=AR\left[x,y\right]+1\Leftrightarrow \left|\left(x_{t}-x_{t-1}\right)x+\left(y_{t}-y_{t-1}\right)y-\frac{x_{t}^{2}-x_{t-1}^{2}}{2}-\frac{x_{t}^{2}-x_{t-1}^{2}}{2}\right|\leq \epsilon$$

Each vector ${\vec{r}}_{t}$ also “votes” for one direction in AT matrix:

(4) $$\forall_{{\vec{r}}_{t}}AT\left[\beta_{t}\right]=AT\left[\beta_{t}\right]+1$$

where: $\beta_{t}=\left\lceil \alpha_{t}\frac{180}{\pi }+180\right\rceil$, $\alpha_{t}=atan2\left(y_{t}-y_{t-1},x_{t}-x_{t-1}\right)$, atan2—means four-quadrant inverse tangent, and ⌈⌉ is ceiling function.

**Figure 7:**
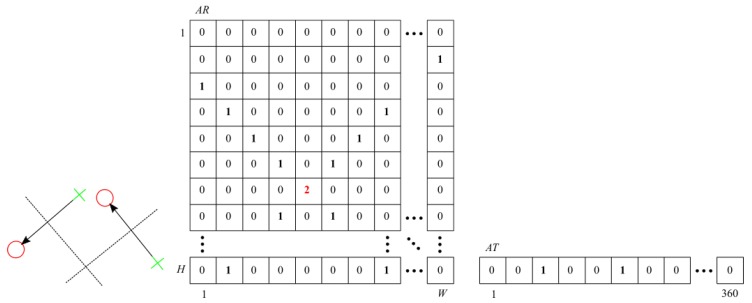
AR and AT after taking into account the “votes” of two pairs of corresponding points.

In the ideal rotation case, all bisectors should intersect at one point in AR (see Figure 7). Due to noise, spatial quantization, and inaccuracy of vision-based measurement, we get a two-dimensional histogram with a maximum.

The decision made by voting reduces the risk that minor faulty matchings not eliminated by statistical analysis will influence the results.

### 2.4. Set of Rules

**Figure 8:**
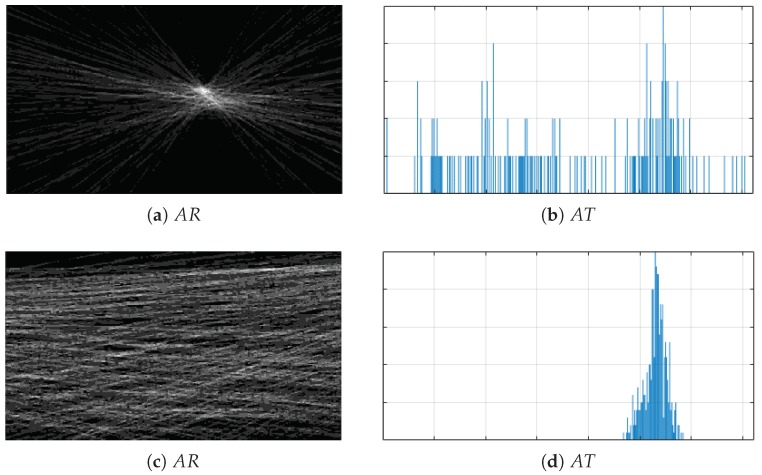
Accumulators: (**a**) AR in rotation, (**b**) AT in rotation, (**c**) AR in recovery, (**d**) AT in recovery.

Figure 8 shows AR and AT accumulator matrices in the rotation (first row) and recovery (second row) phases. The two-dimensional AR matrix was visualized as an intensity image.

AR becomes more “flat” when the rotation of the camera relative to the observed scene decreases and more compact when the rotation is stronger (comp. Figure 8a,c. In the case where the observed scene is dominated by progressive movement (the majority of keypoints moves in one direction), a clear maximum should be visible in the AT histogram (comp. Figure 8b,d).

Therefore, it was proposed to use the histogram spread (HS) measure to determine the plane state [41].

(5) $$HS=\frac{Q_{3}-Q_{1}}{R}$$

where $Q_{1}$ and $Q_{3}$ are the 1st and the 3rd quartile of the histogram and *R* denotes the posible range of histogram values. The 1st and 3rd quartile are the histogram bins at which the cumulative histogram has 25% and 75% of the maximum.

The following set of rules was proposed:

(6) $$Rotation=\left\{\begin{array}{cc} 1 & \mathrm{if}\;HS_{AR}\leq T_{R}\\ 0 & \mathrm{if}\;HS_{AR}\geq T_{R}+\Delta_{R}\\ previous & \mathrm{if}\;T_{R}<HS_{AR}<T_{R}+\Delta_{R} \end{array}\right.$$

(7) $$Recovery=\left\{\begin{array}{cc} 1 & \mathrm{if}\;\left(HS_{AT}\leq T_{T}\right)\wedge \left(\left|arg\;max\left(AT\right)-F\right|\leq \epsilon \right)\\ 0 & \mathrm{if}\;HS_{AT}\geq T_{T}+\Delta_{T}\\ previous & \mathrm{if}\;T_{T}<HS_{AT}<T_{T}+\Delta_{T} \end{array}\right.$$

(8) $$Diving=\left(!Rotation\right)\wedge \left(!Recovery\right)$$

where $T_{R}$, $T_{T}$—threshold values, $\Delta_{R}$, $\Delta_{T}$—deadbands, *F*—the angular value corresponding to the downward movement of keypoints, ϵ—permitted deviation from downward movement. The introduction of dead bands ($\Delta_{R}$, $\Delta_{T}$) prevents short-term state changes when the values $HS_{AR}$ and $HS_{AT}$ oscillate near the threshold values. The method uses the set of rules defined in the parameters domain. That is why it is resistant to local changes in the density of keypoints. Peaks in accumulator matrices also appear when selected parts of the image are devoid of keypoints. It is an analogy to the Hough transform, which can find an analytical description of a curve, also in the case of significant edge discontinuities.

## 3. Experiments

Spin is a dangerous phenomenon. Deliberately performing the spin-entry procedure when testing an experimental method, especially at lower altitudes and with poor visibility, would be extremely risky. Performing some experiments in flight is also impossible because Polish aviation law prohibits aerobatic flights over settlements and other population centers. Therefore, the evaluation of the new approach began with simulation tests, which additionally ensure repeatability of weather conditions.

### 3.1. Laboratory Setup

The X-Plane 10 professional flight simulator was used [42,43]. The simulator operates based on an analytical model of aircraft dynamics and provides images from a virtual camera taking into account geographical location, terrain diversity, time of year and day, cruising altitude, and atmospheric conditions, including visibility. Obtaining data on such diversity under real conditions, in addition to security issues, would also be very expensive. The camera was attached close to the aircraft bow. The experiments were carried out using two computers with the following parameters: Intel Core i7-6700K @ 4 GHz, 64 GB RAM, Nvidia GTX 750 Ti. On one of them, the simulator was launched, on the other, the MATLAB/Simulink computing environment. For the selected conditions, the first flight tests were also performed. Test videos used in the experiments are available at http://vision.kia.prz.edu.pl/.

### 3.2. Dataset

**Figure 9:**
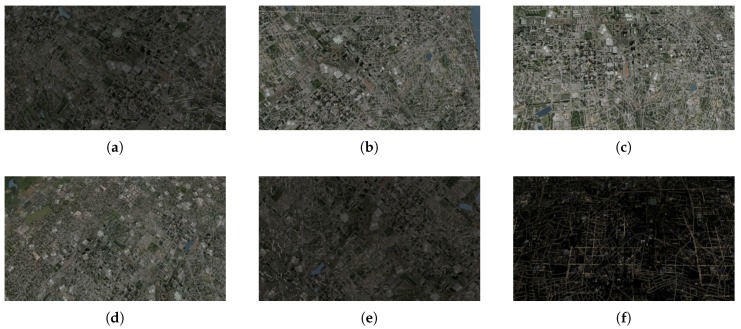
Selected frames from test videos in group 1: (**a**) 6:00, (**b**) 9:00, (**c**) 12:00, (**d**) 15:00, (**e**) 18:00, (**f**) 21:00.

**Figure 10:**
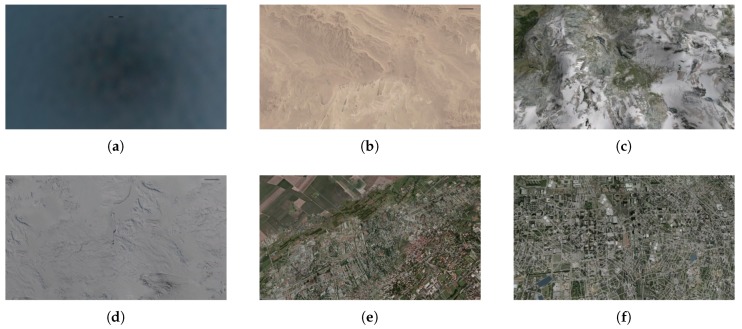
Selected frames from test videos in group 2: (**a**) ocean, (**b**) desert, (**c**) mountains, (**d**) arctic, (**e**) urban1, (**f**) urban2.

**Figure 11:**
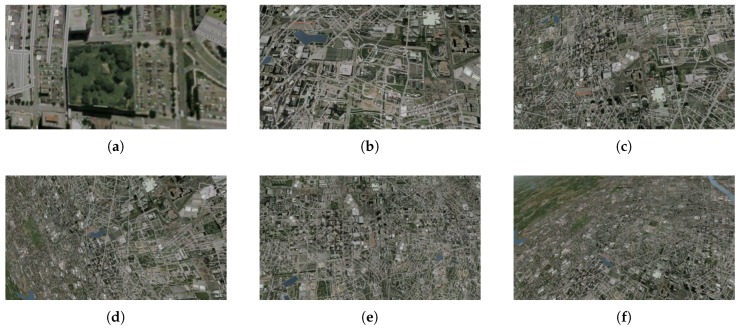
Selected frames from test videos in group 3: (**a**) 2000 ft, (**b**) 4000 ft, (**c**) 6000 ft, (**d**) 8000 ft, (**e**) 10,000 ft, (**f**) 12,000 ft.

**Figure 12:**
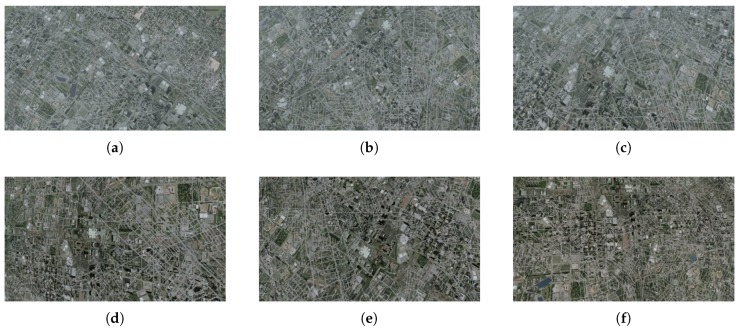
Selected frames from test videos in group 4: (**a**) 0.3 NM, (**b**) 0.4 NM, (**c**) 0.5 NM, (**d**) 3–5 NM, (**e**) 5–10 NM, (**f**) >10 NM.

The dataset consists of 72 test videos divided into four groups (Table 1, Figure 9, Figure 10, Figure 11 and Figure 12). Three recordings were made for each condition.

### 3.3. Results Evaluation Methods

**Figure 13:**
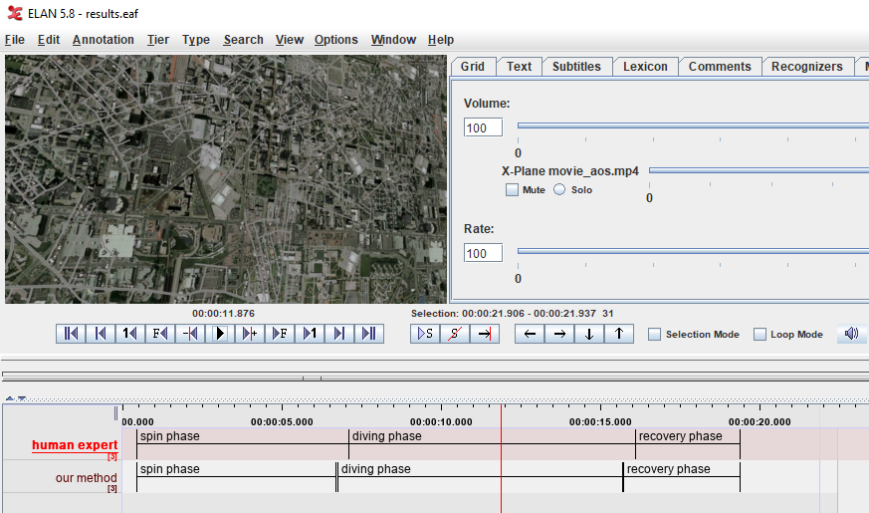
Qualitative evaluation of results in ELAN after automatic insertion of “our method” layer.

Each frame of the manually extracted video fragment corresponding to the entire spin recovery procedure was processed. Manual annotations created by an expert in ELAN—the popular annotation tool were used as a ground truth [44,45]. Results returned by the described method implemented in Matlab were automatically saved in the ELAN file using the annotation API [46]. Qualitative assessment of the results was made by visual comparison of both annotation layers (Figure 13).

For the quantitative assessment, the Jaccard index was used, defined as the length of the intersection divided by the length of the union of ’human expert’ and ’our method’ layers:

(9) $$J\left(A,B\right)=\frac{\left|A\cap B\right|}{\left|A\cup B\right|}=\frac{\left|A\cap B\right|}{\left|A|+|B|-|A\cap B\right|}$$

where *A*—the ground truth (‘human expert’ layer), *B*—the prediction (‘our method’ layer), |A∩B|—length of the layers overlap, and |A∪B|—length of the layers union.

### 3.4. Parameter Selection

The developed method has several parameters characterized in Table 2.

The fixed step size random search (FSSRS) [50] with the fitness function equal to the average Jaccard index, calculated for the entire dataset, was used for parameters selection. The following formula was minimized:

(10) $$f\left(x\right)=-\frac{1}{M}\sum_{i=1}^{M}J_{i}\left(x\right)$$

where $J_{i}$—the Jaccard index (see Equation (9)) estimated for the test video *i*, M=72—number of test videos, and *x*—vector of decision variables composed of method parameters (see Table 2). The initial decision vector $x_{0}$ (a first approximation of method parameters) was selected randomly from the set of allowable values defined in the third column of Table 2. For the first five parameters related to the SURF algorithm, this set was defined based on suggestions given in Mathworks documentation [47,48,49]. For the remaining ones, it was determined experimentally by trial and error approach. The number of steps equals to 100 was proposed as the termination criterion. The lowest obtained value of the fitness function $f_{min}$ was equal to −0.85 for the set of parameter values $x_{min}$ given in Table 3.

### 3.5. Results

The results obtained for the selected parameters are shown in Table 4.

**Figure 14:**
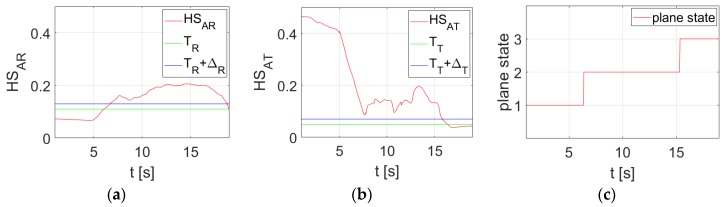
Graphs obtained for the selected test video: (**a**) $HS_{AR}=f\left(t\right)$, (**b**) $HS_{AT}=f\left(t\right)$, (**c**) plane state.

The graphs obtained for the selected test video are shown in Figure 14. In Figure 14a,b, the red lines show the histogram spread measure for *AR* and *AT*, respectively. The green lines correspond to the selected threshold values *T_R_* and *T_T_*. The blue ones show thresholds increased by deadbands *T_R_* + Δ*_R_* and *T_T_* + Δ*T*. Figure 14c shows the state of the aircraft during spin recovery.

For the first group, the Jaccard index was greater or equal to 0.90 in 11 out of 18 cases. This result is promising, given the range of changes in image brightness (compare Figure 9a–f). Moreover, for movies recorded after 21:00, surprisingly good results were noticed, because street and square lighting had a positive effect on the number of keypoints detected. However, they may be worse for areas with less variation in background brightness.

**Figure 15:**
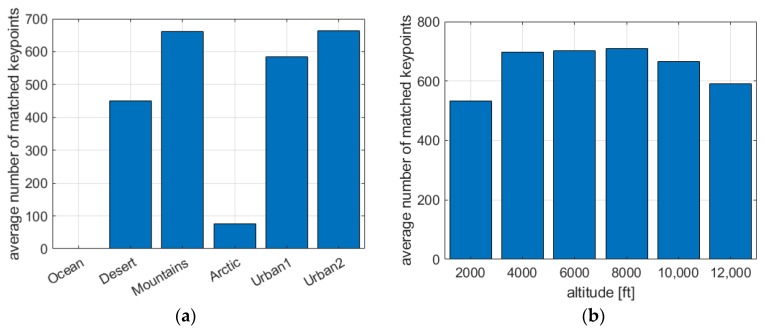
The average number of matched keypoints for: (**a**) different scenes, (**b**) different altitudes.

The results obtained for the second group confirm this hypothesis. It turns out that the method depends on the diversity of the scene. If the spin occurs over areas with a homogeneous structure and small variations in brightness, we get smooth, texture-free images. In such cases, the number of detected and matched keypoints is significantly lower (see Figure 15a). Therefore, the number of votes for the possible rotation center and the dominant displacement direction is also lower. As a result, the method does not infer the real tendency occurring in the processed video accurately. In 3 of 11 cases, the results were weaker (Jaccard index lower than 0.80). For video sequences recorded over a smooth ocean surface, it was impossible to reliably determine the aircraft state due to the small number of matched keypoints. Spin recovery in such background conditions is also problematic for the pilot.

In the third group, some regularity can be seen. The results are weaker for small and large altitudes. At 2000 feet, the objects seen become quite large. The edges and corners between them move apart (see Figure 11a). Because the keypoints are associated with high-frequency elements of the image, their density becomes definitely lower, which results in a lower number of votes (see Figure 15b). At altitudes of 10,000 and 12,000 feet, the edges and corners are so close together that they begin to “merge” into aggregate objects, which also adversely affects the number of detected keypoints. The solution to this problem could be the use of a camera with fast-changing zoom, controlled in an adaptive manner, depending on the height of the aircraft. At such high altitudes, the results can also be affected by the transparency of the atmosphere through which the light beam passes before it reaches the camera lens. (see Figure 11e,f).

**Figure 16:**
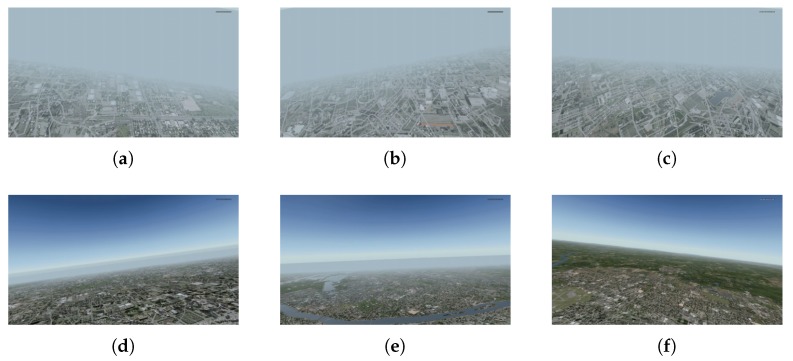
Selected frames from last phase of spin recovery for test videos in group 4: (**a**) 0.3 NM, (**b**) 0.4 NM, (**c**) 0.5 NM, (**d**) 3–5 NM, (**e**) 5–10 NM, (**f**) >10 NM.

Changes in visibility are particularly severe in the last phase of the spin recovery process when the aircraft is in a position close to horizontal (see Figure 16).

The differences in results observed for group 4 are the effect of different lengths of this phase in individual test videos.

**Figure 17:**
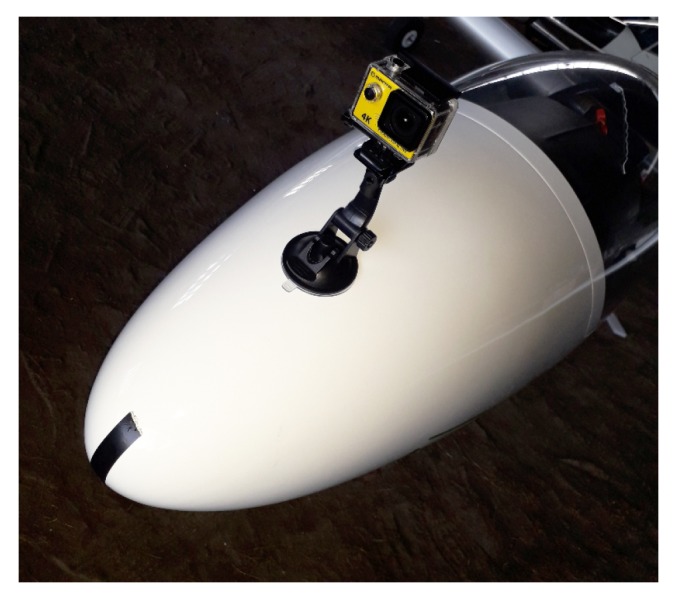
Glider bow with mounted camera used in experiments.

Preliminary experiments for test videos registered during the glider flights were also performed. Flight tests were carried out in September, from 17:00 to 19:00, over the agricultural and forest area, at an altitude of 1500–500 m AGL (Above Ground Level), in CAVOK (Ceiling and Visibility OK) meteorological conditions. The camera was attached to the bow. Its optical axis was approximately parallel to the longitudinal axis of the glider (Figure 17).

Flights were made just before sunset. The sun was low above the horizon, which resulted in rapid changes in image brightness, depending on the spatial orientation of the aircraft, reflections in the lens, and the presence of underexposed areas on the ground due to long shadows. Recorded videos were used to preliminary test the method robustness in adverse lighting conditions.

**Figure 18:**
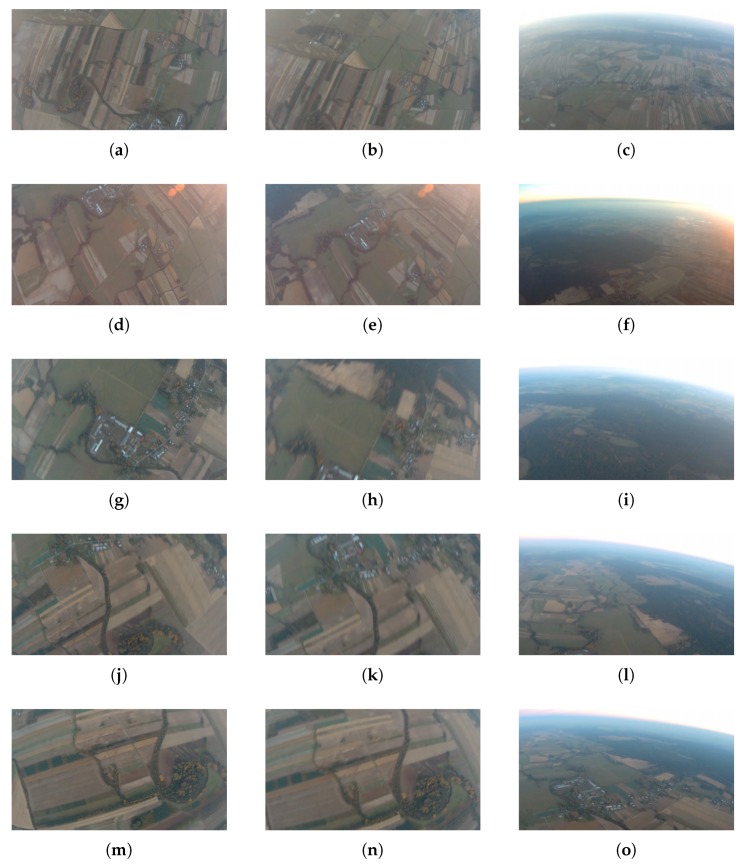
Selected frames from five spin executions: (**a**–**c**) 1st spin, (**d**–**f**) 2nd spin, (**g**–**i**) 3rd spin, (**j**–**l**) 4th spin, (**m**–**o**) 5th spin; columns correspond to spinning, diving and recovery.

Individual rows of Figure 18 show selected frames from consecutive phases of five spin executions.

Table 5 summarizes the results obtained for these videos.

The results obtained for demanding real images recorded on the fly are promising. It turned out that for the execution of the spin during the glider flight, the radius of the spiral line circled by the aircraft is larger. The position of the instantaneous rotation axis, determined by the algorithm, was often outside the image. Therefore, the size of the AR matrix has been doubled. It was also observed that due to the nonuniform scene illumination, the number of keypoints in some parts of the image was too small. The problem was solved by setting the *MetricThreshold* parameter value to 1. The worse results for the first two videos result from the unwanted glares appearing in the lens when it is in full sunlight (see the second row of Figure 18). Perhaps the problem can be solved by using some adaptive image processing algorithms.

In our tests, the single-frame processing time was 250 ms (Matlab), and 30 ms (C++ implementation) for FullHD (1920 × 1080) scaled four times. It is possible to further speed up the calculations by the parallel implementation or the use of an embedded computing system dedicated to vision applications.

## 4. Conclusions

A video-based method to determine the state of the aircraft during the spin recovery process was proposed. It uses the analysis of keypoint shifts in subsequent video frames and the idea of voting on the temporary location of the rotation axis and dominant displacement direction. The decision is based on the set of rules employing the histogram spread measure. Qualitative and quantitative assessments were conducted using a multimedia data annotation tool and the Jaccard index, respectively. The method was validated on the flight simulator videos, recorded at varying altitudes and in different lighting, background, and visibility conditions, as well as the videos acquired during the preliminary flight tests. According to the authors’ best knowledge, this is the first vision-based approach. The results obtained are promising and could be applied in the system supporting the pilot, but further work is needed to achieve the efficiency that would allow the development of a reliable method of automatic spin recovery using vision-based feedback. The following further works are planned: (i) application of adaptive image processing techniques to compensate for non-uniform illumination, (ii) a real-time implementation that would enable online testing during the flight, (iii) tests above the cloud ceiling, (iv) integration of the prepared solution with the horizon detection algorithm, (v) development and testing of the automatic control system.

## Figures and Tables

**Table 1:** Characteristics of the dataset.

Group 1	Description	Change of time of day					
	Goal	Examination of the dependence on the level and direction of lighting					
	Time of day	6:00	9:00	12:00	15:00	18:00	21:00
Group 2	Description	Change of location					
	Goal	Examination of the dependence on the number of detected keypoints					
	Location	ocean	desert	mountains	arctic	urban1	urban2
Group 3	Description	Change of altitude					
	Goal	Examination of the dependence on the number and density of detected keypoints					
	Altitude [ft]	2000	4000	6000	8000	10,000	12,000
Group 4	Description	Change of visibility					
	Goal	Examination of the dependence on the visibility					
	Visibility [NM]	0.3	0.4	0.5	3–5	5–10	>10

**Table 2:** Method parameters.

Parameters Used while Detecting, Extracting and Matching SURF Keypoints		
**Name**	**Description**	**Tested Values**
MetricThreshold	Strongest feature threshold [47]	100, 200, ..., 1500
NumOctaves	Number of octaves [47]	1, 2, 3, 4
NumScaleLevels	Number of scale levels per octave [47]	3, 4, 5, 6
FeatureSize	Length of feature vector [48]	64, 128
MatchThreshold	Matching threshold [49]	1, 10, 20, ... 100
Metric	Feature matching metric [49]	SAD, SSD
**Parameters used while removing faulty matches**		
*k*	Faulty match rejection threshold (Section 2.2, Equation (2))	1, 2, ..., 6
**Parameters used while determining the aircraft state**		
$T_{R}$	Threshold value for $HS_{AR}$ (Section 2.3, Equation (6))	0.05, 0.06, ..., 0.15
$\Delta_{R}$	Deadzone width for $HS_{AR}$ (Section 2.3, Equation (6))	0.01, 0.02, ..., 0.05
$T_{T}$	Threshold value for $HS_{AT}$ (Section 2.3, Equation (7))	0.01, 0.02, ..., 0.10
$\Delta_{T}$	Deadzone width for $HS_{AT}$ (Section 2.3, Equation (7))	0.01, 0.02, ..., 0.05
ϵ	Permitted deviation from keypoints downward movement	0, 5, ..., 45
	(Section 2.3, Equation (7))	

**Table 3:** Selected parameters.

Name	MetricThreshold	NumOctaves	NumScaleLevels	FeatureSize	MatchThreshold	Metric
Value	100	4	6	64	10	SSD
Name	*k*	$T_{R}$	$\Delta_{R}$	$T_{T}$	$\Delta_{T}$	ϵ
Value	1	0.13	0.02	0.10	0.03	40

**Table 4:** The results (Jaccard indices) for X-Plane videos.

Group 1	Time of day	6:00	9:00	12:00	15:00	18:00	21:00
	video 1	0.93	0.88	0.91	0.83	0.91	0.96
	video 2	0.91	0.92	0.94	0.86	0.78	0.91
	video 3	0.96	0.85	0.80	0.93	0.88	0.91
Group 2	Location	ocean	desert	mountains	arctic	urban1	urban2
	video 1	-	0.74	0.99	0.82	0.92	0.91
	video 2	-	0.97	0.91	0.71	0.79	0.96
	video 3	-	0.89	0.99	0.85	0.97	0.80
Group 3	Altitude [ft]	2000	4000	6000	8000	10,000	12,000
	video 1	0.82	0.99	0.94	0.92	0.91	0.75
	video 2	0.79	0.87	0.97	0.90	0.93	0.84
	video 3	0.74	0.90	0.91	0.90	0.77	0.90
Group 4	Visibility [NM]	0.3	0.4	0.5	3–5	5–10	>10
	video 1	0.89	0.82	0.91	0.92	0.82	0.88
	video 2	0.86	0.90	0.88	0.90	0.82	0.93
	video 3	0.76	0.95	0.94	0.89	0.92	0.80

**Table 5:** Results for flight-recorded videos.

**Flight video**	1	2	3	4	5
**Jaccard index**	0.85	0.89	0.92	0.93	0.94

## Abbreviations

The following abbreviations are used in this manuscript:

AOPA	Aircraft Owners and Pilots Association
SIFT	Scale-Invariant Feature Transform
GLOH	Gradient Location and Orientation Histogram
SURF	Speeded Up Robust Features
LESH	Local Energy-based Shape Histogram
HS	Histogram Spread
ft	Feet
NM	Nautical Mile
FSSRS	Fixed Step Size Random Search
AGL	Above Ground Level
CAVOK	Ceiling and Visibility OK
